# FindIT2: an R/Bioconductor package to identify influential transcription factor and targets based on multi-omics data

**DOI:** 10.1186/s12864-022-08506-8

**Published:** 2022-04-07

**Authors:** Guan-Dong Shang, Zhou-Geng Xu, Mu-Chun Wan, Fu-Xiang Wang, Jia-Wei Wang

**Affiliations:** 1 National Key Laboratory of Plant Molecular Genetics (NKLPMG), CAS Center for Excellence in Molecular Plant Sciences, Institute of Plant Physiology and Ecology (SIPPE), Chinese Academy of Sciences (CAS), Shanghai, 200032 China; 2 University of Chinese Academy of Sciences (UCAS), Shanghai, 200032 P. R. China; 3 School of Life Science and Technology, ShanghaiTech University, Shanghai, 201210 China

**Keywords:** Transcription factor, Gene regulation, Chromatin accessibility, ATAC-seq, ChIP-seq, R package

## Abstract

**Background:**

Transcription factors (TFs) play central roles in regulating gene expression. With the rapid growth in the use of high-throughput sequencing methods, there is a need to develop a comprehensive data processing and analyzing framework for inferring influential TFs based on ChIP-seq/ATAC-seq datasets.

**Results:**

Here, we introduce FindIT2 (Find Influential TFs and Targets), an R/Bioconductor package for annotating and processing high-throughput multi-omics data. FindIT2 supports a complete framework for annotating ChIP-seq/ATAC-seq peaks, identifying TF targets by the combination of ChIP-seq and RNA-seq datasets, and inferring influential TFs based on different types of data input. Moreover, benefited from the annotation framework based on Bioconductor, FindIT2 can be applied to any species with genomic annotations, which is particularly useful for the non-model species that are less well-studied.

**Conclusion:**

FindIT2 provides a user-friendly and flexible framework to generate results at different levels according to the richness of the annotation information of user’s species. FindIT2 is compatible with all the operating systems and is released under Artistic-2.0 License. The source code and documents are freely available through Bioconductor (https://bioconductor.org/packages/devel/bioc/html/FindIT2.html).

**Supplementary Information:**

The online version contains supplementary material available at 10.1186/s12864-022-08506-8.

## Background

Gene expression is regulated at different levels. In addition to transcription factors (TFs), the regulatory sequences play an important role in definition of transcriptional competence by integrating multiple cellular or environmental signals. The global regulatory landscape can be inferred by different methods. For example, Chromatin Immuno-Precipitation followed by sequencing (ChIP-seq) measures TF binding and histone modifications at genome-wide level [1]. Assay of Transposase Accessible Chromatin sequencing (ATAC-seq), DNase I hypersensitive sites followed by sequencing (DNase-seq) and MNase digestion followed by sequencing (MNase-seq) can be used to interrogate chromatin accessibility dynamics [2]. Notably, the combination of these methods with transcriptome sequencing (RNA-seq) has become a prevalent strategy for identification of the molecular mechanism and key TFs underlying cell fate determination and developmental trajectory in both animals and plants [3, 4].

The assignment of TF binding sites and the regions with open chromatin or histone modifications (i.e., sequencing peaks) is the prerequisite for data analysis. A set of peak annotation tools such as Homer [5], ChIPpeakAnno [6] and ChIPseeker [7] have been developed. The basic principle for peak assignment is the “nearest gene” strategy, where the algorithm identifies the gene whose transcription start site (TSS) has the closet distance to a given peak. While this principle is acceptable under most conditions [4], it cannot be feasibly applied to the organism with compact genome where peaks reside among multiple protein coding genes. In addition, growing evidence has shown that distal enhancers, i.e., the regulatory cis-elements kilobases or megabases away from the TSS, can also influence gene expression in animals [8, 9]. In particular, an enhancer may have a broad effect on gene expression by regulating more than one gene in its vicinity [10, 11]. As such, there is an urgent need for the optimization of current peak assignment methods.

The quality of TF ChIP-seq dataset is affected by several experimental parameters including the amount of input DNA, the specificity of TF antibody and the enrichment of IP DNAs. One of the solutions to precisely infer the target gene(s) of a given TF is the integration of ChIP-seq peaks with differential gene expression (DGE) data (i.e., perturbed RNA-seq datasets). The traditional integrative analysis approach infers TF targets by taking the intersection of the ChIP-seq target genes revealed by the “nearest gene” principle and differentially expressed genes over an arbitrary threshold. However, as mentioned above, the assignment of ChIP-seq peaks by the “nearest gene” is frequently biased. In addition, an arbitrary threshold will miss some important gene which not show much changes. To address these issues, the software package BETA has been developed [12]. In principle, BETA models regulatory potentials (RPs) for each gene by TF ChIP-seq peaks, and uses rank product to combine the RP result with relevant DGE data [12]. To facilitate its application, a website version of BETA named Cistrome-GO has been recently launched [13]. Unfortunately, BETA and Cistrome-GO only support the analysis of human and mouse genomes. Therefore, the generation of a suitable and universal data processing and analyzing platform for the non-model species is still needed.

Chromatin accessibility inferred by DNase-seq or ATAC-seq aids the identification of regulatory regions in the genome [2]. Given a specific biological process, clustering of all the accessible peaks over time course or across different tissues can yield an overview of regulatory landscape dynamics and delineate stage- or tissue-specific DNA regions associated with cell fate transition and determination. However, how to infer the TFs that regulate a subset of genes or peaks derived from this differential cluster analysis and how to uncover the sequential action and combinatorial activity of TFs are still technically challenging. To address these problems, several bioinformatic tools such as i-cisTarget [14], BRAT [15] and lisa [16] have been developed. However, in order to further increase accuracy, these methods require comprehensive training and integration of a large-scale multi-omics data, thereby hindering its application in less well-studied species.

To address all above issues, we have developed FindIT2, an integrated R package to generate peak-gene pair, infer TF targets and identify influential TFs of query set based on Bioconductor classes and methods [17]. The FindIT2 package can be applied to any species with genomic annotations, and provides flexible and user-friendly functions based on type of data input and analysis purpose.

## Implementation

**Fig. 1:**
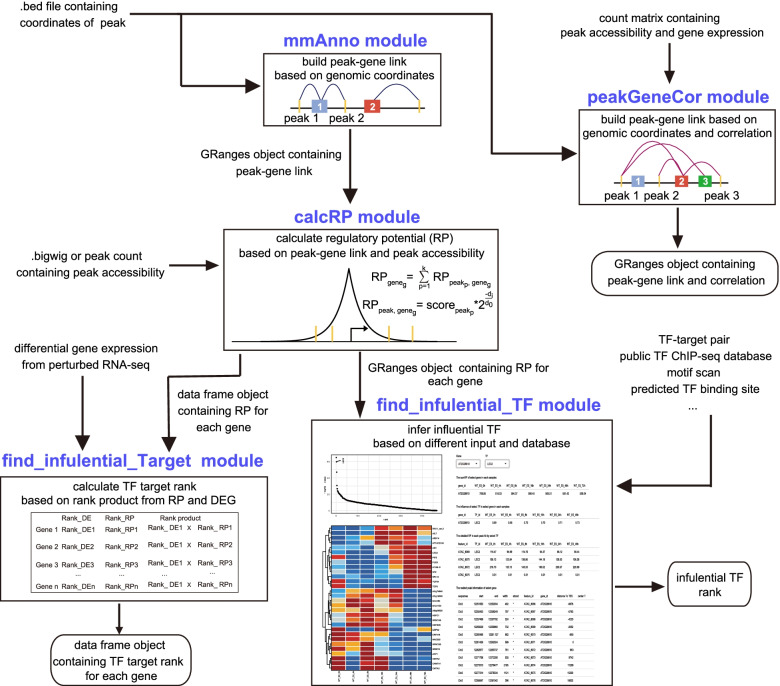
The components of FindIT2. A sketch of FindIT2 components is shown. FindIT2 supports a complete framework for annotating ChIP-seq/ATAC-seq peaks, identifying TF targets by the combination of ChIP-seq and RNA-seq datasets, and inferring influential TFs based on different types of data input. The *mmAnno* module accepts the bed or bed-like format file like narrowPeak, broadPeak which contains coordinates of interesting region. *mmAnno* can build peak-gene links to annotate peak according to the genomic coordinates of features. The *peakGeneCor* module can use the genomic coordinates and count matrix to calculate correlation between features, which can build more robust peak-gene link. The *caclRP* module can accept the peak count matrix and Granges object produced by *mm_geneScan* function in *mmAnno* module to calculate regulatory potential (RP). Or it can also accept bigwig file or TF ChIP-seq peak to calculate RP. The data frame containing RP calculated by *calcRP_TFHit* in *calcRP* module can be integrated with differential gene expression to calculate TF target rank using *integrate_ChIP_RNA* function in *find_influential_Target* module. The *find_influential_TF* provides many methods to infer influential TF based on different analysis purpose and annotation. For example, *findIT_regionRP* can accepts the Granges object from *calcRP_region* to infer influential TF of interesting gene set. *findIT_enrichFisher* can accept public TF ChIP-seq database to find influential TF of interesting peak set

FindIT2 is implemented as an open-source software package using the R programming language, and is compatible with all available operating systems. Most functions in FindIT2 are based on the Bioconductor core methods and classes, which render FindIT2 feasible for non-model species. Instructions on how to install and run FindIT2 are presented on Bioconductor repository (https://bioconductor.org/packages/devel/bioc/html/FindIT2.html). A detailed manual including workflows and operating parameters is given on the Bioconductor page. FindIT2 currently consists of five separate modules. Each module consists of several sub-functions for different input type and analysis purpose (Fig. 1; Table 1). Users can perform specific functions by running these modules separately or build a workflow by the combination of different modules. The overview of FindIT2 and its modules are described below.

**Table 1:** Major FindIT2 functions

Function	Description
loadPeakFile	read peak file and transform it into GRanges object
mm_nearestGene	annotate peaks using nearest gene mode
mm_geneScan	annotate peaks using gene scan mode
mm_geneBound	search related peaks of interesting genes
plot_annoDistance	plot the distance distribution
peakGeneCor	calculate correlation between gene and peak
enhancerPromoterCor	calculate correlation between enhancer and promoter
getAssocPairNumber	get associated peak number of gene and vice verse
plot_peakGeneAlias_summary	plot the distribution of associated feature number
plot_peakGeneCor	plot correlation between two features
shinyParse_peakGeneCor	explore feature relationship interactively
calcRP_coverage	calculate RP using big wig files
calcRP_region	calculate RP based on mm_geneScan and peak count matrix
calcRP_TFHit	calculate RP based on ChIP-Seq peak data
integrate_ChIP_RNA	integrate ChIP-Seq and RNA-Seq data to find TF target genes
findIT_TTPair	find influential TF of input genes based on public TF-target data
findIT_TFHit	find influential TF of input genes based on public ChIP-seq or motif scan
findIT_enrichFisher	find influential TF of input peaks based on public ChIP-seq or motif scan
findIT_enrichWilcox	find influential TF of input peaks based on public ChIP-seq or motif scan
findIT_regionRP	find influential TF of input genes based on RP and public ChIP-seq or motif scan.
findIT_MARA	infer TF activity based on motif scan and peak count matrix
jaccard_findIT_enrichFisher	calculate jaccard index based on findIT_enrichFisher
jaccard_findIT_TTpair	calculate jaccard index based on findIT_TTPair
integrate_replicates	integrate value from replicates

### Multi-peak and multi-gene annotation

The analytic pipeline begins with annotating a region of interest, which is commonly referred to peak annotation. Two annotation strategies, namely “nearest” and “gene scan”, are introduced into the *mmAnno* module in FindIT2 (i.e., the *mm_neaerestGene* and *mm_geneScan* function, respectively). Briefly, the “nearest” strategy identifies the gene whose transcription start site (TSS) has the closest distance to a given peak. The information of corresponding gene is then used to represent peak attribute. Based on this principle, the relationship between peak and gene is one-to-one. As a result, a peak is only annotated once and only linked to one gene. For the “gene scan” strategy, it builds a scan region for each gene and all peaks residing in this region are assigned. The peak not linked to any scan regions is then assigned to the nearest gene. In this scenario, a peak is likely to be assigned to more than one gene.

FindIT2 provides another useful function, *mm_geneBound*, which can be used to identify associated peaks for a gene of interest. This function facilitates the visualization of peak differences on heat maps and volcano plots. The analytic pipeline for *mm_geneBound* starts with using the “nearest gene” strategy to annotate the peaks of interest. For the genes not yet assigned, the “nearest peak” strategy is subsequently applied.

### Calculation of the correlation between peak accessibility and gene expression

Compared with the peak-gene pairing method, the analysis of the correlation of peak accessibility and gene expression can provide more robust association of peaks with the genes that they are predicted to regulate [4, 9]. Based on this assumption, FindIT2 provides the *peakGeneCor* module to calculate correlation scores. The *peakGeneCor* module consists of two functions, namely *peakGeneCor* and *enhancerPromoterCor*. The *peakGeneCor* accepts the peak-gene link results generated by *mm_neaerestGene* or *mm_geneScan*, and uses RNA-seq and ATAC/ChIP-seq peak accessibility values to calculate correlation score and *p*-value. Given the fact that correlation calculation is not robust in a small number of paired samples and most experiment design cannot afford a large number of paired samples, we introduced the *enhancerPromoterCor* function as an alternative strategy. In principle, it considers the nearest peak of each gene as promoter, and calculates the correlation between the distal regulatory elements and promoter. Compared with the *peakGeneCor*, *enhancerPromoterCor* can infer robust association solely based on ATAC/ChIP-seq value. In addition to the above two functions, FindIT2 embeds several other functions including *getAssocPairNumber*, *plot_peakGeneAlias_summary*, *plot_peakGeneCor*, and *shinyParse_peakGeneCor* (a shiny function), which enable users to explore the association of peaks with the genes which they are predicted to regulate.

### Calculation of RP

The RP model [18] is implemented in the *calcRP* module to reconstruct an RP profile for measuring cis-regulatory environment surrounding the TSS of a given gene. The RP score can be applied in the following four scenes. First, it provides a statistic summary of regulatory sequence defined by ATAC-seq/H3K27ac data and serves as a signature for gene expression [19]. Second, the RP score can be used as a maker for identifying cell- or tissue-specific genes based on the ATAC-seq/H3K27ac datasets [19]. Third, it can also represent the confidence level of TF target genes [12] when the RP model is embedded in the TF ChIP-seq data. Finally, after integrating ATAC-seq/H3K27ac data with public TF ChIP-seq or imputed TF binding from motif scan, the RP score can be used to infer influential TFs [16].

To calculate the RP score according to different type of data input and analysis purpose, FindIT2 provides three functions, namely *calcRP_coverage*, *calcRP_region* and *calcRP_TFHit*. The first two functions are designed to process the ATAC-seq/H3K27ac data, while the third function is used for the TF ChIP-seq dataset. The *calcRP_coverage* function calculates the RP score for each gene directly using the ATAC-seq/H3K27ac bigwigfile, whereas the *calcRP_region* uses peak accessibility count matrix file and annotation results from *mm_geneScan* to calculate the RP score. *calcRP_TFHit* accepts TF ChIP-seq peak files generated by call peak tools including MACS2 [20]. The resulting dataset can help users predict direct target genes of a given TF.

### Prediction of TF targets

As mentioned above, the combination of RPs defined by ChIP-seq peaks and DGE analysis improves the inference of direct TF target genes in the model species [13]. However, a general and user-friendly tool for the less well-studied species is currently not available. As such, FindIT2 introduces the *integrate_ChIP_RNA* function to integrate the RP rank results derived from *calcRP_TFHit* and the DGE results generated from diverse RNA differential analysis tools such as DESeq2 [21], edgeR [22], and limma [23]. The *integrate_ChIP_RNA* function is based on rank product [24] which combines RP rank results with DGE. The genes with more adjacent TF binding sites (i.e., ChIP-seq peaks) and higher differential expression ratio are likely to be identified as the targets of high confidence. Users can use this function to infer TF target genes for any species with the TF ChIP-seq and perturbed RNA-seq datasets.

### Inference of regulatory TFs based on different types of data input

Inferring influential TFs involved in a given biological process is a complicated task in comparison to predicting TF targets. Users may want to infer TFs based on genes or peaks of interest, and increase confidence by using different types of public databases. To address these challenges and provide a comprehensive framework for this purpose, FindIT2 introduces the *find_influential_TF* module with six calculation methods. Among them, three methods are designed for input peak set while the other three for input gene set. Meanwhile, peak set and gene set can be converted to each other using the aforementioned *mmAnno* or *peakGeneCor* module, thereby improving flexibility of analysis. In general, these methods meet different analysis purpose and provide different degrees of results according to the richness of annotation of targeted species. Moreover, FindIT2 provides an integrate function, *integrate_replicates*, for users to integrate the results obtained from different source or replicates, thereby increasing the precision of the results.

Identification of enriched TF in a given cluster is the most common analysis purpose. The peak set can be retrieved from different methods such as *k*-means, hierarchical clustering or differential peak expression analysis. To this end, FindIT2 introduces two functions, *findIT_enrichWilcox* and *findIT_enrichFisher*, to reveal enriched TFs by wilcox test or fisher test respectively. A TF with higher number of binding sites in the peak set of interest is likely to be identified as an influential TF. The dataset for the TF ChIP-seq binding sites can be downloaded from public databases such as Cistrome DB [25] and Remap [26]. For the species which do not have public TF ChIP-seq database, the TF binding consensus can be inferred by motif scanning of the ATAC-seq/H3K27ac peak set with the analytic tools including HOMER [5], FIMO [27], and GimmeMotifs [28]. Alternatively, users can predict the TF binding sites using PlantRegMap [29].

Users may be also interested in inferring TFs that regulate a gene set derived from differential, correlated or clustering gene expression analysis. FindIT2 provides two functions for this purpose. The *findIT_TTPair* function fits with the scene where direct TF-target gene database such as RegNetwork [30] and iGRN [31] are available. Similar to the principle of the Gene Ontology (GO) enrichment analysis [32], a TF with multiple direct target genes within a given gene set will be likely to be identified as an influential TF. In contrast, the *findIT_TFHit* function is suitable for the species with public TF ChIP-seq database. A TF with a higher number of binding site surrounding the TSS of a set of genes is likely to be identified. For the species which lacks the TF ChIP-seq databases, users can apply similar strategy as mentioned above.

In addition to the whole get set, users can infer the effect of TF on specific gene or specific peak of a given gene. In this scenario, FindIT2 implements Lisa model [16] into the *findIT_regionRP* function. As a result, the revised function accepts the RP profile results derived from *calcRP_region*, and TF binding site consensus derived from public TF ChIP-seq database or motif scanning. FindIT2 also provides a shiny function, *shinyParse_findIT_regionRP*, to help user explore the impact of inferred TFs on targets interactively.

The Motif Activity Response Analysis (MARA) model [33] is implemented into *findIT_MARA* to reconstruct motif activity trend across several samples. This function is useful when users want to explore timing of TF activities of a given biological process, and can be applied to any species with the TF binding motif dataset.

## Results

To demonstrate the practical utility of the functionalities of FindIT2, we applied it to our recently published datasets related to the chromatin accessibility dynamics during somatic embryogenesis (SE) [34]. We focused on LEAFY COTYLEDON2 (LEC2), a B3-type TF which plays a critical role in SE [34]. We (i) assigned all the ATAC-seq peaks by different annotation modes, (ii) identified direct targets of LEC2 by the combination of ChIP-seq and RNA-seq datasets, (iii) recovered LEC2 as the top influential TF using different methods embedded in FindIT2, (iv) interactively explored the chromatin accessibility of the LEC2 direct targets, and (v) calculated TF activity trend along with SE.

### Assignment of ATAC-seq peaks by different annotation modes

**Fig. 2:**
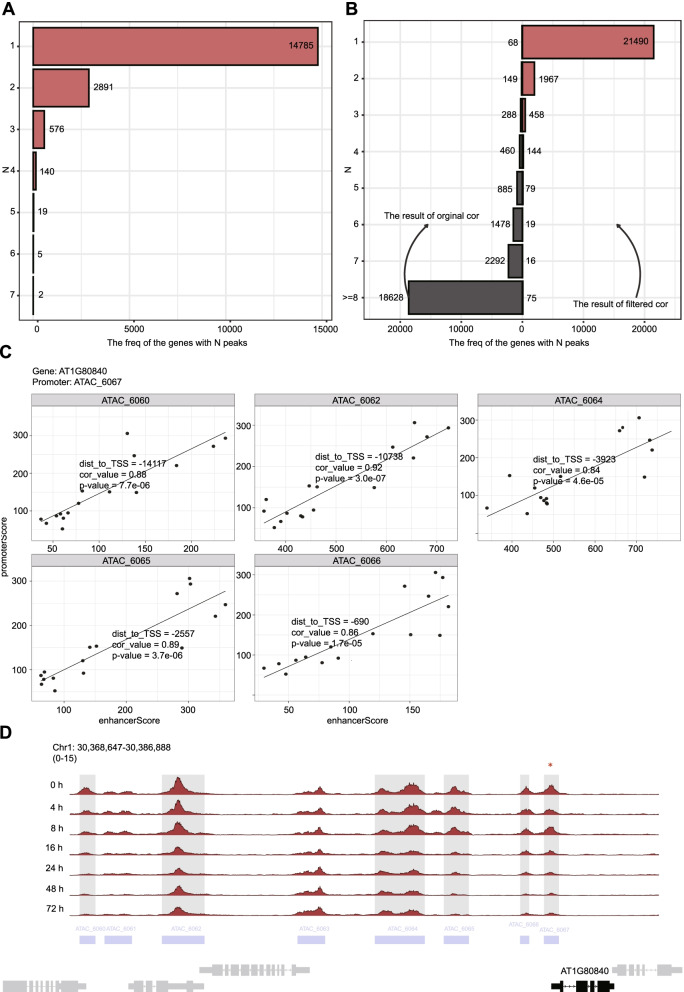
The functional test of the *mmAnno module.* **A** Distribution of the number of peaks linked to a gene inferred by *mm_nearestGene.* The result was plotted by *plot_peakGeneAlias_summay*. **B** Distribution of the number of peaks linked to a gene inferred by *enhancerPromoterCor*. The result was plotted by plot_peakGeneAlias_summay. The origin result is shown on the left. The filtered result is given on the right. Threshold, *p*-value < 0.01 and cor > 0.8. **C** Dot plot of the distal enhancer and promoter accessibility of peak-to-gene link located within 20 kb of AT1G80840. This plot is generated by *plot_peakGeneCor*. **D** The ATAC-seq track of *AT1G80840*. The genomic region is shown and the selected gene is highlighted in black. The locations of the ATAC-seq peaks are indicated by purple rectangles. The related distal enhancer or promoter are shadowed and promoter is marked by an asterisk

**Fig. 3:**
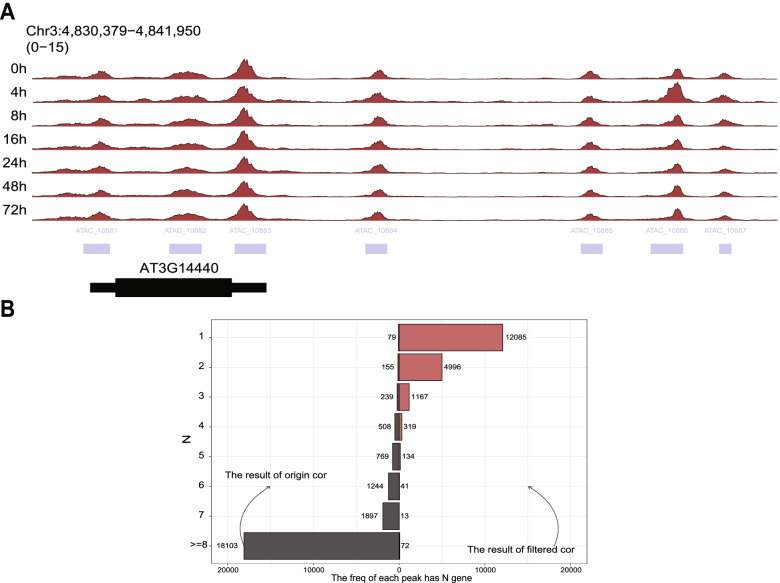
Analysis result of the *AT3G14440* gene locus and *enhancerPromoter* function. **A** The ATAC track of *AT3G14440*. **B** Distribution of the number of genes linked per peak. This result is produced by *enhancerPromoterCor*. The left panel in plot is the origin result, while the right panel is the filtered result according to the threshold: *p*-value < 0.01 and cor > 0.8. This plot is generated by plot_peakGeneAlias_summay

We first illustrated how to use the *mmAnno* module to assign the peaks to the genes which they may regulate. We used the merge peak set derived from the ATAC-seq dataset of the explants at 0, 4, 8, 16, 24, 48, 72 h after induction on E5 media (thereafter named as E5 0h–72h) [34]. We used the *mm_nearestGene* function to annotate peaks and found that, in most cases, each gene is only associated with one peak (Fig. 2A; Additional file 1). One of the genes with 7 peaks is *AT3G14440* (Fig. 3A). *AT3G14440* encodes a 9-cis-epoxycarotenoid dioxygenase, a key enzyme for the biosynthesis of abscisic acid (ABA) in plants. The multiple accessible regions at this gene locus may reflect a complex transcriptional regulatory mechanism and are consistent with the notion that ABA plays a critical role in abiotic stress responses.

We hypothesized that correlation between the accessibility of a distal peak and the promoter of a given gene across different time points denotes a functional connection. The correlation score of each unique link between distal peaks and promoter was calculated by *enhancerPromoter*. Considering the small size of the Arabidopsis genome, we restricted the length of scan region to 20 kb. Using a conservative correlation threshold over 0.8 and *p*-value below 0.01, we identified 4598 unique links between distal peaks and gene promoters (Additional file 2). Most genes have only one related peak and vice versa (Figs. 2B and 3B). However, some genes do have multiple distal peaks. For example, *AT1G80840* (*WRKY40*), which encodes a pathogen-induced TF, harbors five associated distal peaks with its promoter (Fig. 2C and D). Taken together, the above results demonstrate that the “nearest” strategy is feasible under most conditions in Arabidopsis. The correlation information can help users find more useful information.

### Prediction of the direct targets of LEC2

As mentioned earlier, the combination of ChIP-seq and RNA-seq data improves the accuracy for TF targets prediction. We applied the *integrate_ChIP_RNA* function to the LEC2-GR ChIP-seq and RNA-seq datasets [34]. Compared to traditional method that takes the intersection of the ChIP-seq target genes obtained by the “nearest gene” strategy and the differentially expressed genes obtained by the arbitrary threshold, the output of *integrate_ChIP_RNA* provides more detailed information. For instance, users can simultaneously explore TF ChIP-seq and RNA-seq ranking results, thereby facilitating the identification of high-confidence targeted genes (Table 2; Additional file 3).

**Table 2:** The top 10 target genes of LEC2

gene_id	withPeakN	sumRP	RP_rank	log2FoldChange	padj	diff_rank	rankProduct	rankOf_ rankProduct	gene_category	gene symbol
AT2G30470	7	7.026484	2	3.699854	5.27E-64	2	4	1	up	HSI2
AT5G08460	6	7.046663	1	4.554615	3.60E-51	8	8	2	up	NA
AT1G11170	2	3.464122	54	3.26064	2.76E-62	3	162	3	up	NA
AT3G43270	4	2.352608	194	4.30147	1.03E-108	1	194	4	up	NA
AT5G15830	2	3.873782	33	6.779295	1.88E-56	6.5	214.5	5	up	AtbZIP3
AT2G13810	3	5.147604	10	4.602503	1.83E-21	42	420	6	up	ALD1
AT5G23360	5	3.082765	88	3.312275	1.88E-56	6.5	572	7	up	NA
AT5G07550	5	3.759361	36	8.162229	3.32E-36	16	576	8	up	ATGRP19
AT5G57785	6	5.970996	6	5.74865	2.69E-10	100	600	9	up	NA
AT3G59850	2	2.594872	156	4.139314	1.55E-59	4	624	10	up	NA

The “withPeakN” column represents the peak number located in the scan region. The “sumRP” column represents the RP calculated by *calcRP_TFHit*. The “RP_rank” column represents the rank of gene’s RP. The “log2FoldChange” and “padj” columns represent expression fold change and adjust *p*-value, respectively. The “diff_rank” column represents the rank of gene’s padj. The “rankProduct” represents the results of “RP_rank” and “diff_rank”. The “rankOf_rankProduct” represents the rank of “rankProduct” column. The “gene_category” column stands for the gene group according to their expression trend (up, down or static) upon induction of *LEC2*. The “symbol” column represents the gene symbol in the TAIR database. *NA* not available

### Recovering LEC2 as the top influential TF during SE

**Fig. 4:**
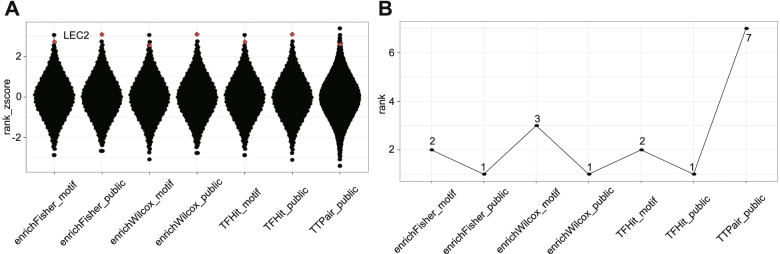
The *findIT_influential_TF* module recovers LEC2 as a top influential TF. **A** The z-score rank distribution of all TFs. The z-score is calculated by converting the rank of each TF into z-scores using inverse normal transformation. LEC2 is marked with red dot. **B** The resultant rank score of LEC2 using each function (X-axis) of the *findIT_influential_TF* module. The ranking results for each function are given

To give an example how the *find_influential_TF* module can be used to identify the influential TFs that regulate a query gene set, we applied this module to the top 1000 LEC2 target genes identified above (Table 2; Additional file 3). To demonstrate that the module can use different annotation types, we used the datasets from two different resources. The first database is the TF binding regions compiled in the Remap2020 [26], and the second is the motif scan results in merge ATAC-seq peak set described above. Importantly, the latter database can be applied to any species with the ATAC-seq datasets. Because the LEC2 ChIP-seq is not available in the Remap database, we imported the LEC2 binding site. As shown in Fig. 4, both *findIT_TTPair* and *findIT_TFHit* successfully ranked LEC2 as one of the most significant TFs among the input genes (Fig. 4; Additional file 4). We also identified FUS3, ABI3, BBM, LEC1, REV, and KAN1 TFs on the top of the list, suggesting that these TFs may cooperatively regulate LEC2 targeted genes. Consistent with this hypothesis, LEC1, BBM, and FUS3 have already been implicated in SE [35].

To test the functions of *findIT_enrichFisher* and *findIT_enrichWilcox*, we used the results derived from *enhancerPromoterCor* analysis. We retrieved the related ATAC-seq peaks and treated these peaks as the input set. By comparing this dataset with total peak dataset, we were able to uncover LEC2 among the top enriched TFs (Fig. 4; Additional file 4).

**Fig. 5:**
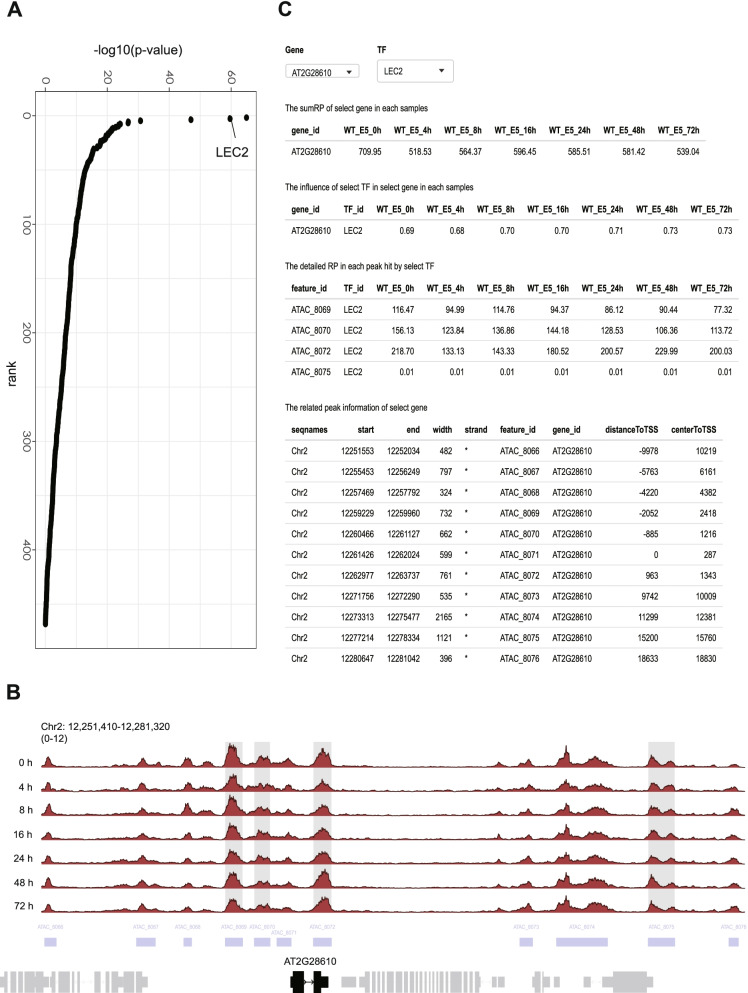
The *findIT_regionRP* function provides detailed information in multi-dimension. **A** The TF ranking result produced from findIT_regionRP. The ATAC-seq dataset at E5 0 h was used. The Y-axis represents the -log10(*p*-value), while the x-axis represents the rank order of all TFs. **B** The ATAC-seq track of *AT2G8610*. The genomic region is shown and selected gene is highlighted in black. The locations of the ATAC-seq peaks are indicated by purple rectangles. The peaks hit by LEC2 are shadowed. **C** The interface of *shinyParse_findIT_regionRP*

The combination of the RP profile with the TF ChIP-seq data can improve the performance of TF inference [16]. We calculated the RPs for each gene during SE with *calcRP_region*. We then used *findIT_regionRP* to identify TFs associated with the top 1000 LEC2 targeted genes and successfully identified LEC2 as one of the top TFs (Fig. 5A). Taken together, these observations indicate that the functions provided by *find_influential_TF* module can be used to infer influential TFs for a given biological process.

### Exploring chromatin accessibilities of the LEC2 direct targets interactively

In addition to identify the influential TFs, *findIT_regionRP* can provide other dimensional information including samples, genes, and features. Users can freely combine multi-dimensional information to extract meaningful results according to their own needs. Meanwhile, FindIT2 provides the *shinyParse_findIT_regionRP* function for users to explore results interactively. For instance, it has been shown that *WOX3* is a direct target of LEC2 [34]. The visualization of datasets by the Integrative Genomics Viewer [36] enables us to reveal that *WOX3* harbors several LEC2 binding sites in the regions which are constantly accessible during SE (Fig. 5B).

Users can further explore the impact of a given TF on specific peak of specific gene with the *shinyParse_findIT_regionRP* function. As shown in Fig. 5C, shiny provides the RPs of *WOX3*, dynamic impact of LEC2 on *WOX3*, detailed information about peak hits by LEC2, and all the ATAC-seq and ChIP-seq peaks surrounding the *WOX3* locus (Fig. 5C). By selecting genes and TFs, users can explore results more quickly and extract more useful information.

### Inference of the timing of TF activities during SE

**Fig. 6:**
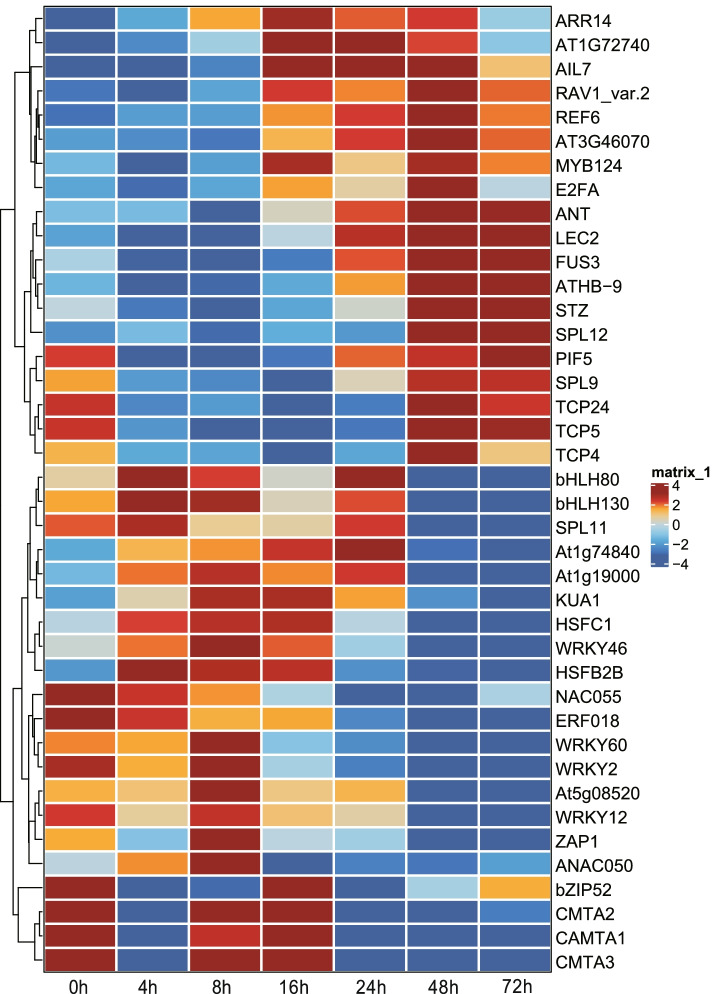
Inference of the timing TF activities during SE by the *findIT_MARA* function. The top 40 highly variable TFs are given. Seven time points along with SE are shown

Calculation of TF variability and dynamic can help users infer potentially important TFs at specific stage during cell fate transitions [3, 4]. We used the *findIT_MARA* function to calculate TF activity trend during SE (Fig. 6; Additional file 5). We found that the WRKY and CAMTA TF binding motifs, which are well-known for their roles in plant immunity, are highly dominant at the early stage of SE. The TFs such as KUA1 and HSFC1 mainly function at middle stage, while the effect of other TFs including ANT, LEC2, FUS3, and ATHB-9 are gradually increased across SE. Overall, all these results are consistent with our published results [34, 37].

## Conclusions

In summary, the above analyses provide a proof-of-concept showing FindIT2 as flexible and powerful tool in dealing with multi-omics datasets. With the popularity of high-throughput ATAC-seq, ChIP-seq, and RNA-seq, we believe that FindIT2 will have a broad application ranging from annotating and processing data to inferring influential TFs and their targets, especially for those non-model species that are less well-studied and lack of high-quality databases.

### Availability and requirements

Project name: FindIT2.

Project home page: https://bioconductor.org/packages/devel/bioc/html/FindIT2.html

Operating system: Platform independent.

Programming language: R.

License: Artistic-2.0 License.

Any restrictions to use by non-academics: none.

## Supplementary Information

**Additional file 1.** The number of related peaks for each gene.

**Additional file 2.** The detailed information of unique links between distal peaks and gene promoters.

**Additional file 3.** The integrated LEC2 target ranking results.

**Additional file 4.** The TF rankings of interesting gene set or peak set.

**Additional file 5.** The TF activity during SE.

## Data Availability

The datasets generated and/or analysed during the current study are available in the GitHub repository https://github.com/shangguandong1996/FindIT2_paper_relatedCode.

## Abbreviations

TF: Transcription factor
ATAC-seq: Assay of Transposase Accessible Chromatin sequencing
DNase-seq: DNase I hypersensitive sites followed by sequencing
MNase-seq: MNase digestion followed by sequencing
TSS: Transcription start site
DGE: Differential gene expression
RPs: Regulatory potentials
GO: Gene Ontology
MARA: Motif Activity Response Analysis
SE: Somatic embryogenesis
LEC2: LEAFY COTYLEDON2
